# Frailty: a tale of two concepts

**DOI:** 10.1186/s12916-015-0420-6

**Published:** 2015-08-11

**Authors:** Jeremy D. Walston, Karen Bandeen-Roche

**Affiliations:** Johns Hopkins University Older Americans Independence Center, Johns Hopkins Medical Institutions, Baltimore, Maryland USA

**Keywords:** Frailty, Frailty index, Frailty phenotype

## Abstract

Frailty is increasingly relevant for clinicians to improve care for vulnerable older adults. Prominent frailty measures include the frailty phenotype and the frailty index. The frailty phenotype is grounded in a theoretical construct hypothesized to have an underlying biological basis. The frailty index describes frailty as a nonspecific age-associated vulnerability, reflected in an accumulation of medical, social, and functional deficits. Building on this model, Minitski et al. describe the development of a biological index that proves to be a reasonable method to predict mortality when compared to other frailty measurements. Strengths include its ability to import clinical measures, interchangeable components, and its potential ability to identify latent risk factors. Obstacles include the lack of a unifying biological theory related to aging, inclusion of costly research measures, and its inability to provide specific clues to the etiology of frailty according to the frailty index definition. Refinement in measures focused on aging-related biological changes rather than using measures that result from chronic disease states could help provide important biological insights and aid in the development of future treatment and preventive modalities.

Please see related article: http://www.biomedcentral.com/1741-7015/13/161.

Frailty in older adults is most often defined as a late-life vulnerability to adverse health outcomes [1, 2]. It has long been an important research topic in geriatric medicine and utilized in research settings to identify at-risk older adults and to study biological underpinnings that drive observed vulnerability. It has more recently gained momentum amongst subspecialists in clinical practice as a method to identify vulnerable individuals undergoing medical or surgical interventions [3].

Although frailty consensus work has focused on physical frailty [1, 2, 4], a theoretically based construct built around a core group of activity-based and strength-based measurements, the frailty index (FI), has emerged as a useful strategy to measure risk for mortality and other adverse health outcomes in older adults. The FI utilizes simple counts of up to 71 co-morbidities, laboratory measures, and social and functional declines (termed deficits) to construct a score [5]. Proponents of this approach have noted that the component measures are interchangeable, the approach can be applied in bedbound or ambulatory populations, and fewer variables can successfully predict mortality than originally proposed [6].

In the present study [7], Mitnitski et al. develop an aging-related biological index that utilizes 40 biological measures found to be age-associated in the Newcastle 85+ study. This index contains measures ranging from telomere length to induced cytokine production from isolated lymphocytes to the components of a complete blood count. Although prior studies of older adults have developed indices focused on clinical laboratory measurements, the authors are to be commended for working toward the development of an index that attempts to assess biological age and associated risk through biological measurements. Their findings regarding the complementarity of clinical and biological measures for predicting mortality risk are potentially important: One can imagine, for example, the identification of older adults who “look” healthy but may benefit from interventions to address significant latent risk factors. Modeling to identify potential modification of risk predicted by each given FI value by the biological measure value could further the two measures’ utility.

There are clear strengths to using an index approach for risk assessment. Any clinical measures included could be abstracted from medical records with minimal effort. The approach is pragmatic in that measures can be for the most part interchanged with other measures without substantial change in the predictive ability of the tools [6]. There are likely a large number of age-related aggregated biological precursors that drive frailty and late-life decline, and the systemic effects as measured by risk to adverse outcomes can in part be detected through an index approach. Hence the approach can be used to predict outcomes, and the combination of FI and a biomarker-based frailty index (FI-B) seems to hold considerable promise to this end. The approach also likely allows the tracking of vulnerability in a reasonable way.

Despite this flexibility in measurement and substantial predictive ability of a high index score, notable obstacles remain if this approach is to be further developed as a true biological aging index or measure of systemic effects. For example, it is not clear that systemic effects are being measured unless index components are selected and validated vis a vis a system. Moreover, including a preponderance of age-associated clinical measures may result in an assessment of chronic disease states rather than aging per se. The FI-B construction, and the authors’ characterization of frailty as “a state of increased risk, compared with others of the same age,” suggests that any marker conferring risk of mortality (or possibly other adverse geriatric outcomes) contributes usefully to frailty measurement. We, and others who consider frailty as a specific physiological state with a definable phenotypic presentation, would disagree. The specificity embedded within the phenotype approach offers benefits if the goal is to elucidate mechanisms and physiological etiology. To approach such a goal scientifically, theories describing plausible processes by which frailty arises, and how they are linked to one another, are needed. Indices (psychometrically: as opposed to “scales”) fail to provide this. Moreover, the more heterogeneously a large collection of variables arises, the higher the risk of masking a key driver that can be targeted by specific interventions. In sum, various conceptualizations of frailty have complementary strengths; however, if etiology is to be elucidated and targeted interventions are to be pursued, we believe the field would benefit from more strongly distinguishing disparate concepts which currently share the single label of “frailty.”

Regarding risk prediction, the current paper’s approach has notable strengths but also a feature mandating caution and further investigation. Among the strengths, definition of biological “deficits” to maximize survival discrimination is highly appealing. Moreover, analysis over random item subsets superbly well elucidates (lack of) sensitivity of predictive accuracy to the choice of specific deficits. However it is important to recognize that the predictive accuracy of the various indices likely has been overestimated, even for older-old populations, because analyses appear not to have been cross-validated (externally or internally, e.g., via a leave-one-out strategy or use of the bootstrap to estimate bias rather than variability [8]. The degree of overestimation could be considerable because of the pre-selection process to maximize survival discrimination in the analytic sample. It will be important to see how the measures perform in external samples, and—as the authors have noted—to evaluate generalization to outcomes other than mortality and a broader age range of older adults.

The FI-B indexing approach as described here could be refined to include a theoretical construct, and tie the choice of measures, around aging biology per se [9]. In addition, to keep the index approach relevant to human subject translation, focus on pragmatic measurements that are relatively easily measured in human subjects must remain a priority in future development rather the complex and costly research measures included in this tool. Despite these critiques, the authors are to be strongly commended for starting a process that could lead to a better understanding of biological aging and how it relates, or contributes, to late-life vulnerability.
